# Preventive Dentistry*Abstract of a paper printed in the Journal of Allied Societies, for June, 1913.

**Published:** 1913-07-15

**Authors:** Henry A. Kelley

**Affiliations:** Portland, Me.


### PREVENTIVE DENTISTRY.*

BY HENRY A. KELLEY, D.M.D., PORTLAND, ME.

If we go back to primitive man and come down through the ages to modern man, we will see that in all this time the endeavor to clean the mouth and teeth has changed but little in theory. Very early in the life of man it seems to have been known that certain foods left upon the teeth caused them to decay, and they used certain tooth powders, brushes and mouth washes to try to prevent this decay, much as most of those who try to prevent decay of the teeth use them to-day. Also, very many years ago it was known that acid substances were in some way unfavorably connected with this decay.

This seemed to give a very easily solved problem, namely, that the way to overcome an undesirable acid condition was by neutralization with an alkali. And so was built up the use of alkaline tooth powders and mouth washes.

In a report to the New York Institute of Stomatology, May 3, 1910, by Alfred P. Lothrop and William J. Gies, it is stated: "The foregoing facts are responsible for our suggestion to Dr. J. Morgan Howe that diluted vinegar or common fruit acids—*acid media ordinarily present in food*—may be very helpful agents in the removal of mucinous masses from the teeth, especially if applied with a suitable instrument. It is probable that the cleansing effect of such treatment would be less harmful to the teeth than the frictional operations now in use for similar purposes." There, in a few words, is the mention of the theory that is, we hope, to lead us on to preventive dentistry.

Gies is amply supported both by Pickerill and Wallace.

It is the consensus of opinion that the way to produce this acid condition, after eating, is by a prescribed diet,

*Abstract of a paper printed in the Journal of Allied Societies, for June, 1913.

though Gies, as quoted, suggested an acid mouth wash and Pickerill also uses one. Gies and his assistants seem to have devoted their researches more definitely to the study of the saliva alone.

Gies and Pickerill have gone pretty well over the whole field of preventive dentistry, and both of these men and their work will be used to back up the position taken by Wallace, to which I wish especially to direct you.

This I do because the subject I wish especially to bring to your attention is the diet, as influencing the decay of the teeth, and the possibility of so influencing and modifying that diet as to make it preventive of dental decay, thus opening the way to a return to the time in the life of man when the percentage of decay was even less than 10 percent., against the more than 90 percent. of to-day. Of course, I recognize that diet alone is not the sole cause of this vast increase of dental caries, but it just happens to be one of the causes, and a very large one, that it is possible we may control.

Other influences, to which I cannot do more than give a mere mention, are the excessive brain work (compared with the muscular), with the resulting indoor life (compared with the outdoor life); the shape of the teeth *themselves*, as well as their arrangement in the jaws (including the shape and size of the jaws themselves), the structural defects of the teeth, the effect of foods upon the *necessity* of mastication and the effect this mastication, if demanded, has upon the size and shape of the jaws, and how this influences the breathing apparatus, and thus the vital force as well as the regularity of the teeth. All this must be passed over.

Miller established, we think beyond question, that in order to induce caries it was first necessary to dissolve out the lime salts of the enamel by the action of an acid, followed by the peptonizing action of the bacteria, the acids being formed from the fermentation of carbohydrate food. Hence, all this endeavor to create an alkaline condition of the mouth and to remove from the enamel these fermentable carbohydrates. We, who have been engaged in this endeavor and have watched the results, know that the results have been unsatisfactory.

J. R. Mummery gives the percentage of skulls with carious teeth in primitive man as follows:

| | |
|---|---|
| Esquimaux | 1.4 |
| Maoris | 3.0 |
| Indians of N. W. American Coast | 3.9 |
| Fiji Islanders | 5.2 |
| Northern Hindoos | 5.9 |
| N. American Indians | 9.5 |
| Eastern Polynesians | 11.4 |
| Southern Hindoos | 14.0 |
| Zulus | 14.2 |
| Sandwich Islanders | 19.0 |
| Australians | 20.5 |
| Bushmen | 20.6 |
| Negroes (Slaves) | 20.8 |

Of course this table is open to modification and is not to be considered as exactly representing the percentage of caries present in each race. but it is sufficiently accurate to compare with our percentage of caries in modren, civilized man, which may be stated roughly as about 90 percent.

I could give you figures to show how civilization of uncivilized man has raised this percentage of dental caries, but the inference is plain.

It is evident there is a distant relationship between the amount of caries and the state of civilization which, it seems to me, is quite the same as saying that our diet and mode of living is the cause of our extensive decay.

If we examine the food we eat and the way we eat it, we will find some very interesting things. First, Wallace found that foods could be divided into two classes—those which tend to leave viscous and fermentable carbohydrates about the teeth, and, second, those which tend to brush them away.

A breakfast, luncheon and supper such as would *not* tend to produce decay is given by Wallace, as follows:

*Breakfast*—Fish, bacon, toast and butter, coffee and tea.

*Luncheon*—Meat or Poultry, potatoes, salad, baked bread, pudding, fresh fruit, water.

*Supper*—Rusks, toast or bread rolls and butter, chicken or fish, an apple, tea or coffee.

And over against this he places a diet which *induces* decay, as follows:

*Breakfast*—Porridge and milk, bread and marmalade. Then perhaps a few hours after a glass of milk and a sweet biscuit (cracker).

*Luncheon*—Mashed potatoes and gravy or minced meat, milk and pudding.

*Supper*—Bread soaked in milk, or bread and jam, cocoa and cake, and a supplementary supper on going to bed of a glass of milk and a biscuit or just "a tiny piece of chocolate."

On comparing these two different types of diet, we observe one is of a kind which stimulates mastication and the last thing taken leaves the mouth clean, or at least free from carbohydrates, so that even when soft food is part of the meal the mouth will be physiologically clean at the end of the meal.

The other type is intended to represent the kind of meal which is calculated to lodge about the teeth and to ruin them within a few years, by making efficient mastication and the self-cleansing of the mouth practically impossible, and by leaving the mouth sticky with fermentable carbohydrates and a virulent crop of acid, forming micro-organisms which have had their development encouraged by the previous meal.

As confirming these recommendations of Wallace, I would submit the following from Pickerill:

Two grammes of each of the substances in the following table were mixed by trituration in a mortar with pestle with 10 c.c. of distilled water, sterilized, infected with 0.2 c.c. of mixed saliva, and incubated for four days. At the end of this time the acid was estimated with the results shown in the table:

| Food Material. Table I. | Acid Units. c.c. of $\frac{N}{5}$ NaOH. | Percentage of Carbohydrates. Hutchinson. |
|---|---|---|
| Chocolate | 5.10 | 57.3 |
| Malted milk (dry) | 4.90 | 70.0 |
| Pastry | 4.10 | 60.0 |
| Toast | 2.40 | 51. + |
| Parsnip | 2.38 | 14.0 |
| Cake | 2.28 | |
| Brown Bread | 2.25 | 48.0 (about) |
| Nuts | 2.16 | 8.5 " |
| Date | 2.00 | 65.7 |
| White bread | 1.87 | 51.5 |
| Milk | 1.76 | 4.5 |
| Biscuit (plain) | 1.65 | 75.0 |
| Cabbage | 1.50 | 5.8 |
| Potato | 1.40 | 19.0 |
| Fig | 1.30 | 62.8 |
| Apple | 1.21 | 12.5 |
| Onion | 0.60 | 6.3 |
| Carrot | 0.35 | 10.0 |
| Apple jam | 0.30 | |
| Raspberry jam | 0.25 | 50.0 (about) |
| Sugar (cane lump) | 0.24 | 100.0 " |
| | $\frac{N}{5}H_2SO_4$ | |
| Fat | — 0.40 | |
| Ham | — 2.45 | |
| Beef | — 2.80 | |

It is seen that all the carbohydrates form some acid, but that there are great differences in the amount formed. For instance, from chocolate is formed more than twenty times as much acid as from cane sugar, and from parsnips twice as much as from apple.

As seen from the second column, these differences do not in the least correspond with the amount of carbohydrates present in each, but rather it depends upon the kind of carbohydrates and its state of comminution.

It is, too, a very significant fact that all those substances from which the least acid is produced are the most tasty substances, and the majority of them, (cabbage, potato, apple, onion, carrot, apple and raspberry jam) are originally acid in reaction. The only materials which gave an alkaline reaction were the proteins—beef, ham and fat.

The question which now arises is: Can we, by a combination or sequence of salivary depressants and excitants, reduce the fermentable debris remaining in the mouth after a meal?

*Effect of sequence*—Substances which were known to produce most acid were eaten and then followed immediately by other substances which had been shown to result in an alkaline reaction. The teeth were then brushed and the mouth rinsed and the washings incubated, with the following results:

| Substance. | Alkaline Units. |
|---|---|
| Bread and butter followed by apple | Neutral |
| Bread and butter followed by orange | Neutral |
| Bread followed by radish | 0.15 (alkaline) |
| Bread followed by fish | 0.03 (alkaline) |
| Bread followed by duck | 0.10 (alkaline) |
| Rice followed by orange | 0.20 (alkaline) |
| Chocolate followed by apple | 0.20 (alkaline) |
| Cake followed by orange | 0.15 (alkaline) |

Here, then, is an important fact, that by such a sequence the deleterious effects of acid-forming substances can be completely overcome and an acid result transformed into a neutral or alkaline one. This, obviously, is of the utmost importance as a physiological means for the prevention of caries, and offers a most simple and efficacious method whereby *the commencement of the process of decay may be obviated.*

A *mixture* of these substances, while showing a like result, was not nearly so efficacious as the *sequence.*

Therefore, to prevent the retention of fermentable carbohydrates on and between the teeth, and so eliminate or very considerably reduce the carbohydrate factor in the production of decay, starches and sugars should on no account ever be eaten alone, but should in all cases either be combined with a substance having a distinctly acid taste, or they should be followed by such substances as have been shown to have an "alkaline potential," and the best of these are, undoubtedly, the natural organic acids found in fruits and vegetables.

Next, it was observed by Wallace that whenever a set of teeth were seen in which the amount of decay was but little, that one of two things was true. Either the person did not eat the foods that tend to leave viscous and fermentable carbohydrates about the teeth, or if these foods are eaten the meal is finished with the foods that tended to brush them away. This we would call the mechanical cleaning of the teeth by the action of mastication.

But, of course, there is much more to it than that. After this arrangement of the food so that at least the last article of the meal is detergent, we will consider what part the saliva plays in this prevention of decay. The much discussed question of the sulphocyanate of potassium and its ability to retard or prevent decay I must not consider. It is rather to consider the *cleansing properties* of the saliva and not its antiseptic properties, if such it has, for we believe it is the fermenting carbohydrates *held for long periods* against the enamel that cause the enamel to break down and allow decay to commence.

If we could have our saliva as we desire, we would choose to have first enough in amount to act as a wash to dissolve and float off from the teeth food particles capable of dissolution and removal in this way. Second, to be alkaline enough to overcome the acids of the mouth, either introduced as acids or the acids formed by the decomposition of foods.

I must exclude the questions of amount of ptyaline, sulphocyanate, potassium phosphate, chlorides, etc., and their effects.

Can the diet influence the amount and the alkalinity of the saliva? Most certainly.

Pickerill says: "It is evident that the rate of flow must be extremely important in considering the mechanical cleansing of the mouth and teeth by the water of the saliva alone, and the dilution of the acids formed by fermentation. The greater the flow per minute, the more rapidly will the acids be carried away. It is always a question of time. The organisms form a certain amount of acid from carbohydrate in a certain time, and it is a question whether the saliva can dissolve and carry away the carbohydrate debris or neutralize those acids as they are formed, or whether the acid production gets ahead of the saliva and is free to combine with the lime salts of the enamel."

The amount of saliva can be stimulated or depressed. The stimulants are conveyed via the optic, olfactory or auditory nerves. Of foods, the most tasteful substances, and especially if they are acid as well as tasty—i. e., orange, apple, carrot (raw), figs, stewed apple and lemon. Among the salivary depressants are soft and little flavored foods and tannic acid (tea). Sodium carbonate shows a markedly depressant effect, and chalk, soap and carbonate of soda used with the tooth brush caused a lowering of the c.c. per minute from the normal 1.65 to 1.00, while the use of acid potassium tartrate (powder) showed an increase in c.c. per minute from normal 1.65 to 1.90.

We thus see the amount of the saliva is very important and is completely under control *as to amount.*

*Alkalinity*—The most *acid* substances produce the greatest alkalinity. As compared with neutral substances, they are as 11 : 1. Orange, apple, pineapple, celery, carrot and lemon are foods which markedly raise the alkalinity of the saliva.

Thus we see that the acid forms of foods are the ones that increase the amount of saliva and likewise increase the alkalinity of the saliva. *Depressors* of alkalinity are sweet substances—chocolate, cake, grapes, bananas, figs, etc., bread and butter, brown bread, meat and biscuit (crackers).

This increase of alkalinity of the saliva by the use of an acid diet seems at first as paradoxical, but a little consideration, supported by experiment, proves it *true.* If we take a mouth that is neutral or slightly acid and wash it out with a quite strongly alkaline substance (mouth wash) it will, of course, show at once a marked increase of alkalinity, but unfortunately this increase of alkalinity is very fleeting, and in a very few minutes—two or three—the saliva will return to normal and most often to subnormal alkalinity. It is most probable that a constant use of an alkaline mouth wash will in time cause a permanent loss both in alkalinity and totality of saliva. (Pickerill's experiments prove this to be so.) And this is the very thing we have been doing with our tooth powders and mouth washes.

TABLE VI.—PICKERILL.

Total Saliva Five Minutes After Use of Dentifrice.

| Substances used as Dentifrice | C.c per Minute. | Alkalinity per c.c. | Alkalinity per Minute |
|---|---|---|---|
| Normal saliva (resting) to show increase or decrease | 1.65 | 1.05 | 1.73 |
| Chalk, soap and carbonate of soda with tooth brush | 1.00 | 0.90 | 0.90 |
| The same used with finger, traces remaining in the mouth | 1.10 | 1.30 | 1.43 |
| Chalk, carbonate of soda, cloves and gaultheria | 1.30 | 1.00 | 1.30 |
| Acid potassium tartrate (powder). | 1.90 | 1.20 | 2.28 |

We cannot but conclude from this that the use of alkaline dentifrices for the prevention of caries is wrong, is physiologically incorrect, unscientific and empirical; and not only so, but also actually conducive to the inception and progress of disease, by decreasing the circulation and alkalinity of fluids in the mouth.

The use of alkalies seems to be based upon a wrong conception. It is as though it were thought that lactic acid developed and accumulated in the mouth, remaining there for some hours, or until next morning, when an overwhelmingly strong alkali is introduced to neutralize it; whereas, of course, as each molecule of lactic acid is formed, it searches for something wherewith to combine. Alkaline salts of the saliva will obviously most readily satisfy it, but should these not be available, then the calcium phosphates and carbonates of the enamel surface are utilized.

It cannot be too clearly recognized that, by the use of alkalies, only those molecules of acid formed immediately previously can be neutralized, and also that the natural defensive forces of the mouth are thereby lowered for some considerable time afterwards.

On the other hand, if a weak solution of a fruit acid, the vinegar of Gies or the citric or tartaric acids of Pickerill, or the fruits themselves as recommended in the diet by Wallace, be used, of course there is at once a marked increase in the acidity of the saliva, but this gives way to a *marked* increase in the alkalinity in two or three minutes, and this alkalinity lasts at least for fifteen minutes.

It is only when an acid remains upon the tooth, unneutralized and unsatisfied, for a long period, that harm is done. There is, therefore, no expectation or evidence that this weak fruit acid can or will do harm, nor should there be any expectation that an alkaline reaction lasting two or three minutes, as from the alkaline mouth washes and tooth powders, will be efficacious in overcoming the formations of acids of decomposing foods.

The acids to us in this treatment must be strong enough to *stimulate* the mechanism controlling salivary secretion and yet not so strong as to defeat the desired end. Pickerill suggests tartaric acid as per formula:

R

| | |
|---|---|
| Potass tart ac | gr. ii |
| Ac tartarici | gr. i |
| Ol limonis | m. iii |
| Glusidi | gr. ¼ |
| Aqua | ad. ℥i |

I do not see how I could better present this to you than this quotation from Pickerill:

"1. It is evident the saliva is a fluid extremely variable in its composition and amount, but that these variations do not occur without reason, but rather in obedience to fixed and definite laws and in response to certain ascertainable stimuli.

"2. The mechanism controlling salivary secretion is extremely sensitive and complex, since different 'flavors' of little intensity are capable of being 'selected' and give rise to secretions of saliva differing widely in character and amount.

"3. That practically all the normal constituents of saliva are, if present in sufficient amount, of value and importance in protecting the teeth against the occurrence of dental caries, and in maintaining the health of the oral mucous membrane.

"4. That *acids*, and particularly the 'natural' organic acids, are stimulants which excite the greatest amount of these protective substances per minute, and, moreover, give rise immediately, and for a considerable time afterwards, to an increased *alkalinity* of the mouth. That, conversely, substances of little or no distinctive 'flavor' and also alkalies, produce a diminution in the amount of protective substances per minute, and reduce the alkalinity of the mouth both at once and for some time afterwards.

"5. That in the saliva is provided a natural and potentially perfect mouth wash acting continuously day and night (not merely for a few minutes a day). THAT IT IS, MOREOVER, COMPLETELY UNDER CONTROL; THAT IT MAY BE ALTERED OR VARIED IN AMOUNT OR COMPOSITION; THAT ITS BENEFICIAL EFFECTS MAY BE INCREASED OR DECREASED ABSOLUTELY AT WILL."

We have thus seen something of what we would like to do and that there is strong probability we can do it. Should I not say there is *proof*, for what else mean these words quoted above of Pickerill and Wallace, words uttered in no uncertain tone?

If these observations be correct, then it should be possible to so develop a child that it should be quite free from dental caries.

Wallace says: "It now only remains to bring forward even more startling evidence, and to make it more obvious to those who did not or would not take the trouble to follow the facts and arguments which demonstrate the truth of the theory. It appeared to me the most satisfactory way of doing this would be to get people with infant children to put the theory into practice. Fourteen children have been subjected to the test, and at ages ranging from five to seven years their teeth were examined, with the result that not one tooth of any of these children showed the slightest trace of caries. This may not seem a large number, but when we remember that in England a similar number of children of the same class would certainly have had eighty or ninety carious teeth among them at the same age, we see that according even to this alone there is overwhelming probability or practical proof that the theory is correct."

